# Time-dependent effects of CX3CR1 in a mouse model of mild traumatic brain injury

**DOI:** 10.1186/s12974-015-0386-5

**Published:** 2015-09-02

**Authors:** Heidi Y. Febinger, Hannah E. Thomasy, Maria N. Pavlova, Kristyn M. Ringgold, Paulien R. Barf, Amrita M. George, Jenna N. Grillo, Adam D. Bachstetter, Jenny A. Garcia, Astrid E. Cardona, Mark R. Opp, Carmelina Gemma

**Affiliations:** Department of Anesthesiology and Pain Medicine, University of Washington, BOX # 359724, Seattle, WA 98001 USA; Neuroscience Graduate Program, University of Washington, Seattle, WA 98104 USA; Department of Biology, University of Washington, Seattle, WA 98104 USA; Sanders-Brown Center on Aging, University of Kentucky, Lexington, KY 40536 USA; Department of Biology and South Texas Center for Emerging Infectious Diseases, University of Texas at San Antonio, San Antonio, TX 78249 USA; Present address: Interdepartmental Program in Neuroscience, University of Utah School of Medicine, Salt Lake City, Utah USA

**Keywords:** CX3CR1, TBI, Microglia, Cognitive function, Cytokines

## Abstract

**Background:**

Neuroinflammation is an important secondary mechanism that is a key mediator of the long-term consequences of neuronal injury that occur in traumatic brain injury (TBI). Microglia are highly plastic cells with dual roles in neuronal injury and recovery. Recent studies suggest that the chemokine fractalkine (CX3CL1, *FKN*) mediates neural/microglial interactions via its sole receptor CX3CR1. CX3CL1/CX3CR1 signaling modulates microglia activation, and depending upon the type and time of injury, either protects or exacerbates neurological diseases.

**Methods:**

In this study, mice deficient in CX3CR1 were subjected to mild controlled cortical impact injury (CCI), a model of TBI. We evaluated the effects of genetic deletion of CX3CR1 on histopathology, cell death/survival, microglia activation, and cognitive function for 30 days post-injury.

**Results:**

During the acute post-injury period (24 h–15 days), motor deficits, cell death, and neuronal cell loss were more profound in injured wild-type than in CX3CR1^−/−^ mice. In contrast, during the chronic period of 30 days post-TBI, injured CX3CR1^−/−^ mice exhibited greater cognitive dysfunction and increased neuronal death than wild-type mice. The protective and deleterious effects of CX3CR1 were associated with changes in microglia phenotypes; during the acute phase CX3CR1^−/−^ mice showed a predominant anti-inflammatory M2 microglial response, with increased expression of Ym1, CD206, and TGFβ. In contrast, increased M1 phenotypic microglia markers, Marco, and CD68 were predominant at 30 days post-TBI.

**Conclusion:**

Collectively, these novel data demonstrate a time-dependent role for CX3CL1/CX3CR1 signaling after TBI and suggest that the acute and chronic responses to mild TBI are modulated in part by distinct microglia phenotypes.

## Background

Traumatic brain injury (TBI) is a serious public health problem in the United States. Mild TBI is rarely recognized in a timely manner because symptoms often do not manifest for some time after the injury. However, the consequences of mild TBI can be long lasting. The pathophysiology of TBI is complex, in part because multiple pathways are involved in the primary and secondary injuries that result from the insult.

A consensus based on recent evidence considers neuroinflammation as an important secondary mechanism that plays a role in delaying injury after TBI [1–3]. Dysregulated and uncontrolled microglial activation may be a key component of chronic neuroinflammatory processes. Recent studies suggest that the chemokine fractalkine (CX3CL1, *FKN*) mediates neural/microglial interactions via its sole receptor CX3CR1. Indeed, deleting CX3CR1 [4] exacerbates microglial neurotoxicity induced by systemic inflammation in rodent models of Parkinson’s disease and amyotrophic lateral sclerosis [5]. In addition, CX3CR1^−/−^ microglia exacerbate cell-autonomous microglial neurotoxicity induced by lipopolysaccharide (LPS), suggesting that fractalkine signaling is important for limiting microglia toxicity [5, 6]. CX3CL1 also protects against excitotoxicity through the activation of the ERK1/2 and PI3K/Akt pathways [7, 8]. However, the role of FKN/CX3CR1 signaling in neurodegenerative disease is an intricate and highly debated research topic that is becoming even more complicated as new studies reveal seemingly discordant results. It appears that FKN/CX3CR1 signaling plays a direct role in neurodegeneration and/or neuroprotection depending upon the CNS insult. Both beneficial and detrimental effects of CX3CR1 deficiency are associated with microglia activation [9–11]. Data from some models of chronic neuronal injury suggest that CX3CR1 is protective because the absence of this receptor increases microglia activity and is associated with less favorable outcome [8, 12–16]. For instance, in a model of Alzheimer’s disease (AD), CX3CR1-deficient microglia from mice overexpressing hTau have increased hyperphosphorylated tau and more toxicity, as has been observed in other animal’s disease models [5, 17–20]. On the other hand, in 3x-Tg AD mice, which reproduces both Aβ and tau pathologies, deleting CX3CR1 prevents neuronal loss and microglial migration without affecting amyloid deposition [21]. Finally, other reports indicate that CX3CR1 deficiency attenuates amyloid deposition in AD mouse models characterized by extensive plaque deposition [18, 22].

The complex CX3CL1/CX3CR1 signaling pathway is further complicated by its functional role in acute injuries. Results from some acute injury models suggest that CX3CR1 is detrimental because its absence is associated with a favorable outcome. For instance, in a model of cerebral ischemia and spinal cord injury, CX3CR1^−/−^ mice showed neuroprotection and improved functional recovery [23–26]. These observations suggest that CX3CR1 may play different roles in neuronal injury depending on the type of injury and on the post-injury time point assessed (chronic vs acute). CX3CL1 levels increase in the cerebrospinal fluid and in the brain of patients with head trauma, and increased CX3CL1/CX3CR1 is associated with better clinical outcome [27, 28]. However, few pre-clinical studies of TBI focus on the CX3CL1/CX3CR1 system. Determining a role for CX3CL1/CX3CR1 signaling in outcomes after mild TBI during the acute and chronic phase is a goal of the present study.

We now know that the microglia assume diverse phenotypes in response to specific microenvironmental signals. Although there is a spectrum of different microglial types, there are two major microglia phenotypes characterized by a molecular signature of gene expression: the “classical” inflammatory state (M1) and the “alternative” activation state (M2). M1 microglia are generally considered pro-inflammatory whereas, M2 microglia are generally anti-inflammatory [29–34]. In the healthy young brain, M1 and M2 microglia exist in a state of dynamic equilibrium. Following an injury, there is a shift into an M1 phenotype that exacerbates tissue injury or into an M2 phenotype that promotes CNS repair, depending on the local signals in the lesion’s microenvironment [35].

In this study, we demonstrate that lack of CX3CL1/CX3CR1 signaling in the brain leads to delayed neuronal damage and neurologic and cognitive impairment after TBI that is associated with a switch in microglia phenotype. Our data suggest a time-dependent effect of CX3CR1 on modulating brain injury and suggests a potential unique therapeutic window for treating TBI neuroinflammation.

## Materials and methods

### Animals

All experiments were conducted in accordance with the National Institute of Health Guide on Care and Use of Laboratory Animals and were approved by the Institutional Animal Care and Use Committee of the University of Washington. CX3CR1^−/−^ (*CX3CR1*^*GFP/GFP*^) mice were obtained from The Jackson Laboratory (Bar Harbor, Maine) and a colony established at the University of Washington. A total of 180 3-month-old male CX3CR1^−/−^ and littermate CX3CR1^+/+^ (wild-type) mice were used in these experiments. Mice were group-housed in environmentally controlled conditions (12:12 h light-to-dark cycle at 21 ± 1 °C) and provided with food and water *ad libitum*. After surgery, animals were single-housed.

### Traumatic brain injury

Adult mice were anesthetized with isoflurane (induced at 4 %, maintained at 1.25–1.5 %) and placed in a stereotaxic frame equipped with a heating pad to maintain body temperature. A midline incision was made, fascia removed, and a 5-mm diameter craniotomy was made over the left parietal cortex between the lambda and bregma. The bone flap was removed and the integrity of the underlying dura assessed. TBI was induced using a controlled cortical impact (CCI) device (Leica Impact One, Richmond, IL, USA) equipped with an electrically driven 3-mm diameter metal piston controlled by a linear velocity displacement transducer. Impact velocity was set at 5.0 m/s, with a dwell time of 100 ms, and a depth of 0.5 mm from the dura. Previous studies have reported that these parameters produce a mild injury [36, 37]. A 5-mm disk created from a polystyrene weighing boat was glued to the skull over the craniotomy, and the incision closed with sutures. Mice were kept on a heating pad and allowed to recover until ambulatory before being returned to their home cages. Sham (uninjured control) animals received identical anesthesia and craniotomy but were not subjected to CCI brain injury.

### Tissue collection and processing

For immunohistochemistry studies, animals were anesthetized with isofluorane and transcardially perfused with ice-cold phosphate-buffered saline (PBS), followed by 4 % paraformaldehyde in PBS. The brains were postfixed in 4 % paraformaldehyde for 24 h, after which they were transferred to a 30 % sucrose in PBS for at least 16 h at 4 °C. Coronal sections of the left hemisphere were made at 40 μm using a cryostat (Leica CM1950) and stored in cryoprotectant at 4 °C. Animals used for protein assessment were euthanatized by rapid decapitation. Brain regions of interest were dissected and rapidly frozen in liquid nitrogen before storage at −80 °C. Subsequently, both the hippocampi and the sections of cortex were homogenized using an electric tissue homogenizer in 1:10 weight-to-volume ratio of ice-cold RIPA buffer (Millipore; Billerica, MA, USA) containing protease inhibitors and EDTA (Pierce; Rockford, IL, USA). Following homogenization, sample lysates were centrifuged at 10,000×*g* at 4 °C for 15 min and the supernatant collected.

### Biochemical endpoints

#### Enzyme-linked immunosorbent assay (ELISA)

The total protein in each sample was determined using the bicinchoninc acid method (BCA; Pierce Biotechnology, Inc.).

#### Analysis of gene expression by qRT-PCR

Dissected cortex tissues from adult male mice were stored at −80 °C, and total RNA was isolated with RNeasy Lipid Tissue Mini Kit (QIAGEN, catalog # 74804) with on-column DNase treatment (QIAGEN, catalog # 79254) according to the manufacturer’s protocol. RNA quantity and quality were determined using A260/A280 readings by a NanoDrop (ThermoScientific) spectrophotometer. Reverse transcription (RT) was performed using High Capacity cDNA Reverse Transcription Kit Assay (Applied Biosystems, catalog # 4368814) following the manufacturer’s protocol. “No template” and “no RT” controls were included in all assays.

RT-PCR was performed using the TaqMan Gene Expression Assay (Applied Biosystems, catalog # 4331182) according to the manufacturer’s instructions on a 7300 Real Time PCR System (Applied Biosystems). The following TaqMan probes were used: Il-1β (Mm00434228_m1), Cd86 (Mm0444543_m1), Fcgr1 (Mm0438874_m1), Tgfb1 (Mm01178820_m1), Arg1 (Mm00475988_m1), Nos2 (iNOS), (Mm00440502_m1), and 18S (Mm03928990_g1). Relative gene expression was calculated by 2^−ΔΔCT^ method.

### Immunohistochemistry

Except where indicated, staining was done on 40-μm free-floating sections of a one-in-six series through the entire hippocampus and cortex. Tissue was blocked in 10 % normal serum from the species in which the secondary antibody was raised, with the addition of 0.1 % Triton X-100. Primary antibodies were diluted in 3 % normal serum with 0.1 % Triton X-100, and tissue incubated overnight at 4 °C. Biotinylated secondary antibodies were diluted in 3 % normal serum with 0.1 % Triton X-100 and were incubated for 2 h at room temperature. Enzyme detection was done using avidin-biotin substrate (ABC kit, Vector Laboratories, Burlingame, CA, USA) followed by color development in diaminobenzidine solution (Sigma-Aldrich, St. Louis, MO, USA). Antibodies and dilutions were as follows: rat CD11b (Serotec, Raleigh, NC, USA; 1:500); mouse CD68 (Serotec; 1:200); rabbit YM1 (Stem cell technology, Vancouver, BC, Canada; 1:400); rat Marco (Serotec; 1:500); mouse NeuN (Millipore, Temecula, CA, USA; 1:1000). After incubation for 24–48 h with appropriate primary and biotinylated secondary antibodies, tissue was treated with Vectastain ABC reagent (Vector Labs) and visualized with DAB reaction (Sigma-Aldrich). *Controls included omitting primary or secondary  antibodies.*

### Fluoro-Jade B staining

Fluoro-Jade B staining was performed according to Kubova et al. [38]. Briefly, 40-μm coronal free-floating sections were washed three times for 10 min with 0.1 M phosphate buffer saline (PBS), mounted on glass slides, and dried overnight at 37 °C. The sections were then incubated in chloroform-ethanol (1:1) for 1 h. Next, tissue was rehydrated in absolute ethanol (3 min), 70 % ethanol (2 min), distilled water (2 min), incubated in 0.06 % KMnO_4_ for 15 min, rinsed in distilled water (2 min), and incubated for 30 min in a solution containing 0.001 % Fluoro-Jade B (Histo-Chem, Jefferson, AR, USA) and 0.1 % acetic acid. Sections were then rinsed in distilled water, dehydrated in a series of xylene and coverslipped.

### Stereological analysis

All cell counts were obtained using the Optical Fractionator method of unbiased stereological counting techniques [39]. An Olympus BX-51 microscope and the Stereo Investigator software (MBF Bioscience, Colchester, VT, USA) were used to obtain cell counts on sections systematically sampled throughout the entire hippocampus and parietal cortex as described earlier [40]. Neuroanatomical borders of the hilus of the dentate gyrus were outlined, and counting was confined to these areas. The virtual grid (175 × 175) and counting frame (100 × 100) were optimized to count at least 200 cells per animal with error coefficients less than 0.07. Outlines of the anatomical structures were made using a 10×/0.45 objective, and cell quantification was done with a 60×/1.40 objective.

### Isolation of mononuclear cells and flow cytometry

Perfused brains were dissected from mice 15 and 30 days post-TBI, mononuclear cells separated over discontinuous 70/30 % percoll gradients as previously described [41, 42], and cellular pellets resuspended in cell staining buffer (Biolegend, San Diego, CA, USA). Blood for single stained controls was collected from the submandibular vein and RBCs depleted by hypotonic lysis and washed in staining buffer. Isolated cells were incubated on ice for 5 min with anti-mouse CD16/CD32 (clone 2.4G2; BD Pharmingen) to block Fc fragment receptor (FcR)s and then incubated on ice for 30 min with a mix of fluorochrome-conjugated anti-mouse Abs; CD45-APC-Cy7 or APC (Clone 30-F11, BD Pharmingen), CD11b-APC or PerCP-Cy5.5 (Clone M1/70), CD11c-PeCy7 (Clone N418, eBioscience), Ly6G-PE (Clone 1A8, Biolegend), and I-A/I-E-PerCP or Pacific Blue (Mouse MHC-II Clone M5/114.15.2). After washes, cells were resuspended in 2 % paraformaldehyde and analyzed in a LSR-II (BD Biosciences, Franklin Lakes, NJ, USA). To quantify the proportion of resident and infiltrating myeloid cells undergoing cell proliferation, CNS mononuclear cells were stained first with cell surface markers, fixed with 4 % PFA for 30 min and then incubated at RT for 10 min in permeabilization buffer (eBioscience). Cells were then stained with Ki67-V450 (Clone B56) in permeabilization buffer for 20 min, washed, resuspended in 2 % PFA, and acquired on an LSR-II [43].

### Behavioral analysis

#### Composite neuroscore

Evaluation of neuromotor impairment following CCI was performed by using a 28-point composite neuroscore. In brief, mice were evaluated by an investigator blinded to the injury status (sham, TBI) of the animal. Scoring for each animal ranged from 0 (severely impaired) to 4 (normal strength or function) for each of the following modalities: (1) left and (2) right forelimb flexion during suspension by the tail; (3) left and (4) right hind limb flexion with the forelimbs remaining on a flat surface as the hind limbs are lifted by the tail; (5) ability to resist lateral pulsation to the left and (6) right; and (7) ability to stand on an inclined plane in the left, (8) right, and (9) vertical position. In addition, the ability of mice to stand on an inclined plane for at least 5 s in each of three directions (facing up, left, and right) was determined. The angle was increased in 2.5° increments starting at 40°; the maximal angle was noted and compared with pre-injury (baseline) values. A score was then assigned based on the decrease from baseline angle in degrees, where 4 = 0° change, 3 = 2.5° change, 2 = 5° change, 1 = 7.5° change, and 0 = 10° change or greater. Each of the three directions was scored separately, after which a mean was calculated, giving a maximum score of 4 for the inclined plane. The scores for (7), (8), and (9) were averaged, and a composite neurological motor score (0–28) was calculated by summation of individual test scores. Neuromotor function was assessed in both injured and sham-operated animals, and baseline composite neuromotor scores were calculated from values obtained 24 h prior to injury.

#### Open-field and elevated plus maze

The open-field and elevated plus maze were used to determine general activity levels and to measure anxiety-like behavior. Animals were monitored under moderate lighting for 15 min in a 40-cm^2^ open field using video tracking software (ANY-Maze, Stoelting, IL, USA). General activity was evaluated by determining the total of distance traveled. Anxiety-like behavior was assessed based on the pattern of exploration in the open field (center versus periphery). Anxiety-like behavior was also assessed using the elevated plus maze. Our elevated plus maze consists of two well-lit open arms (35 cm) facing each other and two enclosed arms (30.5 cm) also facing each other. Each arm is attached to a common center platform (4.5 cm^2^), and the entire apparatus was elevated 40 cm off the floor. The mice were placed in the center platform and allowed to explore for 5 min. Video tracking software (ANY-Maze) measured movement and time spent in each section.

#### Rota-rod

Mice were tested for overall balance, motor coordination, and motor learning on an accelerating rota-rod apparatus (Ugo Basile, Italy). The center rotor is a 3-cm diameter cylinder, and rotation started at 4 rpm and accelerated to 40 rpm within a 5-min period. Mice were tested for the time spent on the rotor during each of four trials with a 30-min inter-trial interval, and time to fall off the rotor was the outcome measured.

#### Morris water maze

The Morris hidden platform water maze (MWM) consisted of a circular pool (1.38 m diameter) filled with room temperature water containing non-toxic opaque paint with an escape platform (10 cm diameter) hidden 3 cm beneath the surface. Each mouse was placed in the pool in a pseudorandom order and given 60 s to locate the escape platform. When the mouse found the platform, or if it failed to find the platform within 60 s, it was placed on the platform and left for 30 s. Each mouse was given four trials per day for 7 days with 1-h inter-trial interval between trials. Sessions were recorded, and the time to find the platform (escape latency), the total distance traveled, and swim speed were determined by video tracking software (ANY-Maze). After each session, mice were towel-dried and placed in a cage on a heating pad until dry, after which they were returned to their home cage. On day 8 post-training, all mice were subjected to one probe trial in which the platform was removed and each animal had 60 s to search the pool for the platform.

### Statistical analysis

Statistical analyses comparing two genotypes, two manipulations (sham and TBI), and two time points (15 and 30 days post-injury) were done using a one-way analysis of variance (ANOVA) or two-way ANOVA with post hoc multiple comparison analysis (Tukey-Kramer or Bonferroni post hoc test). Repeated measures ANOVA followed by Bonferroni post hoc comparisons were used to analyze rota-rod data. Flow cytometry data are presented as number of cells or percentage of specific cell populations. Differences between groups were analyzed using ANOVA or an unpaired *t* test with GraphPad Prism software (San Diego, CA, USA). *P* values are shown in the data (*) when *P* < 0.05. Statistical analysis was done in the GraphPad Prism software.

## Results

### Mild TBI impairs neuromotor function

**Fig. 1:**
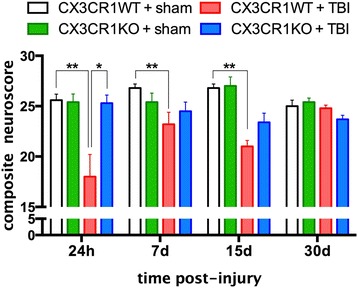
Composite neuroscore (mean ± SEM). Evaluation of neuromotor function and recovery over a 30-day period. At 24 h post-TBI, injured WT mice (*n* = 3) demonstrated a significant decline in neuromotor function compared with sham WT (*n* = 4) and CX3CR1^−/−^ injured mice (*n* = 4; two-way ANOVA, *F* (1, 9) **p = 0.001; **p = 0.001). At 7 days post-TBI, injured WT mice (*n* = 5) continued to demonstrate significant impairment than sham WT mice (*n* = 5; two-way ANOVA, *F* (1, 15) *p = 0.01). Injured CX3CR1^−/−^ mice (*n* = 4) were not different from the injured WT mice (*p* > 0.05). A significant impairment in motor function performance was still present at 15 days post-injury in injured WT mice (*n* = 8) compared to sham WT mice (*n* = 8; two-way ANOVA, *F* (1, 15) **p = 0.001;). Injured CX3CR1^−/−^ mice (*n* = 8) were not different from the injured WT mice (*n* = 8; *p* > 0.05). All injured mice (*n* = 5) recovered similarly at 30 days post-TBI. *White bar* = CX3CR1 sham WT. *Green bar* = CX3CR1 sham KO. *Red Bar* = CX3CR1 WT-TBI. *Blue bar* = CX3CR1 KO-TBI

Prior to surgery, there were no differences in composite neuroscores between WT and CX3CR1^−/−^ mice. The brain-injured WT mice had lower composite neuroscores at 24 h, 7, and 15 days post-surgery than sham WT mice (Fig. 1). By 30 days post-surgery, composite neuroscores for the brain-injured WT and sham WT mice did not differ, indicating recovery of neuromotor function. Composite neuroscores for the brain-injured WT mice were significantly lower than those of brain-injured CX3CR1^−/−^ mice at 24 h post-injury but not at 7 or 15 days post-injury. Composite neuroscores for the brain-injured CX3CR1^−/−^ mice did not differ from those of sham CX3CR1^−/−^ mice at any time point, indicating that CX3CR1 deficiency is neuroprotective.

**Table 1:** Total microglia cell numbers in the brain tissue. Microglia cell numbers in sham WT, sham KO, WT-TBI, and KO-TBI at 15 and 30 days post-TBI as plotted in Fig. 4b. Total microglia cell numbers in the brain tissue. Microglia cell numbers in sham WT, sham KO, WT-TBI, and KO-TBI at 15 and 30 days post-TBI as plotted in Fig. 4f

	15 days				30 days			
	Sham WT	Sham KO	WT-TBI	KO-TBI	Sham WT	Sham KO	WT-TBI	KO-TBI
Total cell numbers in the brain tissue after TBI (Fig. 4b)								
Mean	390,777	984,242	1.581 × 10^6^	1.255 × 10^6^	1.408 × 10^6^	1.697 × 10^6^	1.617 × 10^6^	1.986 × 10^6^
Standard error	90,972	120,144	389,298	254,594	302,104	222,274	301,655	137,642
*n*	6	6	*6*	6	8	7	9	8
Microglia cell numbers in the brain tissue after TBI (Fig. 4f)								
Mean	216,305	377,880	468,937	471,776	487,667	885,237	701,797	862,206
Standard error	46,031	56,377	84,401	73,242	79,322	156,709	142,253	128,469
*n*	6	6	6	6	8	7	9	8

### Mild TBI does not affect spontaneous locomotor activity or cause excessive anxiety but impairs motor learning and cognitive function

Separate groups of animals (*n* = 10/group) were evaluated in open-field, elevated plus maze, rota-rod, and Morris water maze 30 days post-TBI.

To examine spontaneous locomotor activity in response to a novel environment, all mice were tested in the open-field behavioral task. The open-field task monitors activity in a brightly lit, novel environment. Spontaneous locomotor activity was assessed as the total amount of distance traveled in the chamber. In addition, anxiety levels can be measured in the open-field task through assessment of the distances traveled in the center versus perimeter of the chamber. This task exploits the natural tendency of mice to avoid open areas.

**Fig. 2:**
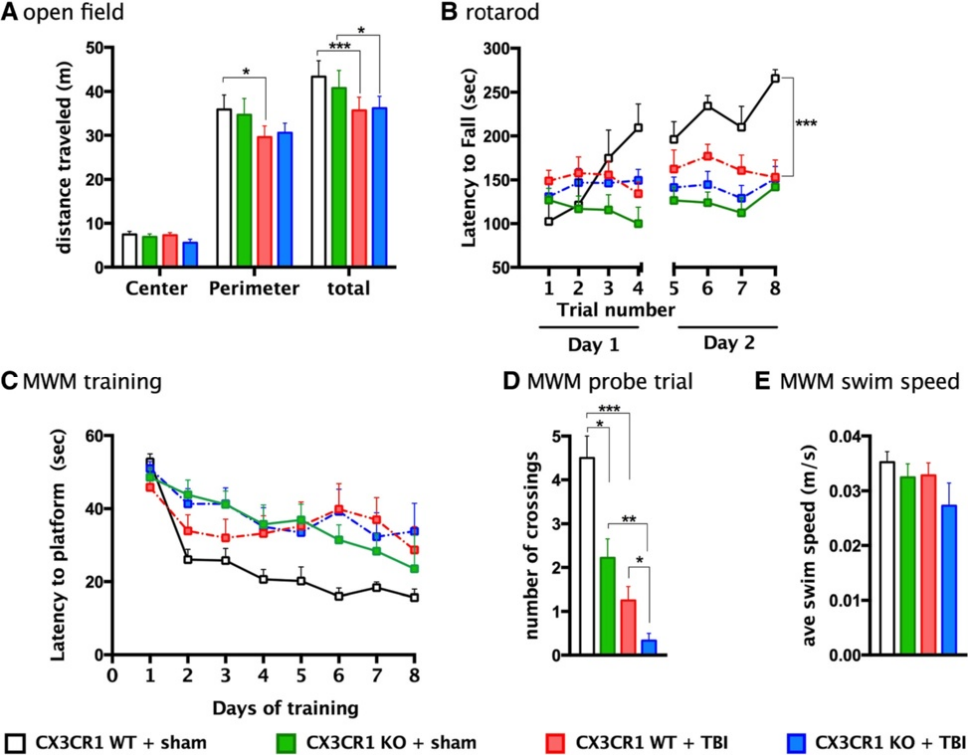
Evaluation of behavioral analysis 30 days post-TBI. **a** In the open-field task, all mice traveled the same distance in the center zone compared to the perimeter zone. CX3CR1 WT-TBI spent less time in the perimeter zone of the open-field chamber compared to CX3CR1 KO-TBI (two-way ANOVA (*F* = 3, 72, **p = 0.01). Total distance traveled in the chamber was significantly different between CX3CR1 sham WT and CX3CR1 sham KO mice. Two-way ANOVA (*F* = 1, 36, **p = 0.001) and between shams and TBI groups two-way ANOVA (*F* = 1, 36, **p = 0.001). CX3CR1 sham WT (*n* = 10 *white bar*), CX3CR1 sham KO (*n* = 10 *green bar*), CX3CR1 WT-TBI (*n* = 10 *red bar*), and CX3CR1 KO-TBI (*n* = 10 *blue bar*) mice. **b** Rota-rod. CX3CR1 sham WT mice performed significantly better than CX3CR1 sham KO mice, over the 2 days of training (repeated measures ANOVA, *p = 0.5). No difference between injured groups was observed in the learning ability in the rota-rod task during the first day of training. On the second day of training (trials 5–8), CX3CR1 sham WT mice learned the rota-rod task as demonstrated by their ability to remain on the rod for longer periods compared to CX3CR1 WT-TBI (**p = 0.01). CX3CR1 KO-TBI mice showed a significantly worse performance than CX3CR1 WT-TBI wild-type (*p = 0.5). Neither the CX3CR1 KO-TBI nor the CX3CR1 WT-TBI mice showed significant improvement in motor coordination with training when compared to sham WT. CX3CR1 sham WT (*n* = 10; *white open square*). CX3CR1 WT-TBI (*n* = 10; *red square*). CX3CR1 sham KO (*n* = 10; *green square*). CX3CR1 KO-TBI (*n* = 10; *blue squares*). **c**–**e** Morris water maze. **c** Mean latency to escape from a pool to a hidden platform across training days. All groups spent more time in learning to find the platform compared to sham WT. CX3CR1 sham WT (*n* = 8; *white bar*). CX3CR1 WT-TBI (*n* = 9; *red square*). CX3CR1 sham KO (*n* = 8; *green square*). CX3CR1 KO-TBI (*n* = 9; *blue squares*). **d** A probe test was performed on day 8 to determine the number of pseudo platform crossings in the target quadrant. All TBI groups crossed less number of time the target quadrant zone when compared to CX3CR1 sham WT. CX3CR1 KO-TBI mice crossed the target quadrant less number of time than CX3CR1 sham KO and CX3CR1 WT-TBI. **e** No difference was observed in the average swim speed between groups. CX3CR1 sham WT (*n* = 8; *white bar*). CX3CR1 WT-TBI (*n* = 9; *red square*). CX3CR1 sham KO (*n*= 8; *green square*). CX3CR1 KO-TBI (*n* = 9; *blue squares*). All data are presented as mean ± SEM *p* < 0.01

In the open-field task, there were no genotype differences in distance traveled. WT mice subjected to TBI spent less time in the perimeter zone of the open-field chamber than did CX3CR1^−/−^-injured (TBI) mice (two-way ANOVA **p = 0.01; Fig. 2a). A modest, yet statistically significant difference in total distance traveled was revealed between sham and TBI groups for both genotypes; however, no difference was observed between WT-TBI and CX3CR1^−/−^ TBI mice. No difference was revealed between genotypes and/or injury groups in the elevated plus maze task (data not shown).

After open-field and elevated plus maze assessments, the same mice were also tested for coordination and motor skill acquisition using an accelerating rota-rod during each of four trials per day on two consecutive days (Fig. 2b). The amount of time an animal stays on the rota-rod is an indicator of its general level of balance and coordination. In general, mice improve their performance over time with training, which is an indicator of motor learning. Sham wild-type mice performed significantly better than sham CX3CR1^−/−^ mice, during the 2 days of training (Fig. 2b), confirming our previous finding of motor learning deficits in CX3CR1^−/−^ mice [40]. No differences were observed among injury groups during the first day of training. On the second day of training (trials 5–8), sham WT mice remained on the rotor for longer periods. Injured CX3CR1^−/−^ mice performed worse than injured WT. However, the motor impairment of injured CX3CR1^−/−^ mice was similar to that one observed in sham CX3CR1^−/−^, likely due to the baseline (floor) effect associated with CX3CR1 deficiency. Neither injured CX3CR1^−/−^ mice nor injured WT mice significantly improved motor coordination on the second day of training when compared to day 1 of training; (Fig. 2b; repeated measures ANOVA, *p* < 0.0001).

Spatial learning was evaluated using the Morris water maze (MWM) task as previously described [40]. As previously shown, the escape latency (time to find the platform) of sham CX3CR1^−/−^ mice during training was longer than that of sham WT mice (Fig. 2c). All mice subjected to TBI, irrespective of genotype, took longer to find the platform during training as compared to sham WT mice. During the probe trial (day 8), the number of target platform crossings was decreased in injured mice irrespective of genotype as compared to sham WT mice. Injured CX3CR1^−/−^ mice performed worse than injured WT and sham mice (Fig. 2d). A similar trend was observed in the time that each group spent in the platform zone (data not shown). There was no difference in the average swim speed among groups (Fig. 2e).

### TBI-induced neuronal cell death and survival

**Fig. 3:**
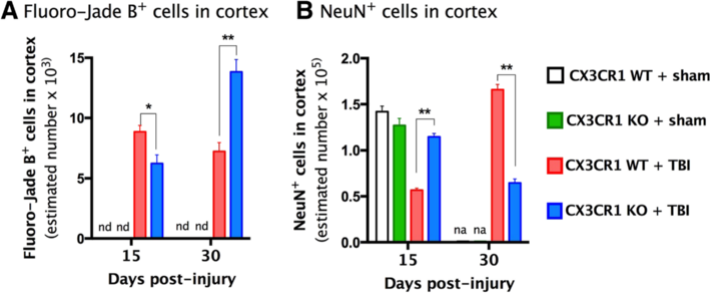
Quantification of neuronal loss at 15 and 30 days post-TBI. *Left* panel **(a)**: unbiased stereology quantification showed a significant decrease in the number of Fluoro-Jade B^+^ cells in the ipsilateral cortex (*F* = 1, 10; *p = 0.001) of CX3CR1 KO-TBI mice (*n* = 3; *blue bar*) at 15 days post-TBI, when compared to CX3CR1 WT-TBI (*n* = 3; *red bar*). CX3CR1 sham WT (not detected), CX3CR1 sham KO (not detected). Fifteen days post-TBI, the decrease in the number of Fluoro-Jade B^+^ cells in CX3CR1 KO-TBI mice was associated with a significant increase in the number of NeuN^+^ cells (*right pane* **(b)**; *n* = 3) compared to CX3CR1 WT-TBI (*n* = 3 **p = 0.001). Thirty days post-TBI CX3CR1 KO mice showed increased number of Fluoro-Jade B^+^ cells (*n* = 3; *p = 0.01) which was associated with decreased NeuN immunoreactivity (*n* = 5; **p = 0.001; mean ± SE (*WT vs CX3CR1^−/−^ ± 15 vs 30 days). CX3CR1 sham WT (*white bar*), CX3CR1 sham KO (*green bar*), CX3CR1 WT-TBI (*red bar*), and CX3CR1 KO-TBI (*blue bar*). At 30 days, the number of NeuN^+^ cells was not determined (nd). Two-way ANOVA performed across genotype, treatment, and time point revealed a significant difference in the number of Fluoro-Jade and NeuN^+^ cells when compared between 15 and 30 days post-injury

We used Fluoro-Jade B and NeuN staining to determine the impact of mild TBI on neuronal cell death and survival in the cortex during the transition from the acute to chronic post-traumatic period, days 15 and 30 post-TBI. Stereological analyses revealed increased numbers of degenerating neurons (Fluoro-Jade B^+^ cells) in the cortex of WT mice 15 days post-TBI as compared to CX3CR1^−/−^ mice (Fig. 3a; two way ANOVA, *p* = 0.0001; Bonferroni post hoc *p =* 0.01). By 30 days post-injury, however, the number of degenerating neurons in injured CX3CR1^−/−^ mice was greater than in injured WT mice (Fig. 3, *p* = 0.001). Stereological quantification revealed that the number of Fluoro-Jade B^+^ cells in CX3CR1^−/−^ mice increased from day 15 to 30 post-TBI, suggesting delayed cell degeneration in these animals (CX3CR1^−/−^-TBI—15 vs 30 days, *p* < 0.01). Microscope visualization did not reveal any Fluoro-Jade B^+^ cells in sham animals irrespective of genotype (nd).

Neuronal survival was determined by the stereological quantification of NeuN^+^ cells (Fig. 3b). At 15 days post-TBI time point, we analyzed tissue from all groups of animals (CX3CR1 sham KO, CX3CR1 sham WT, CX3CR1 KO-TBI, CX3CR1 WT-TBI). However, at 30 days, only tissue from injured TBI animals was analyzed. Neuronal cell loss was apparent after TBI in WT mice and CX3CR1^−/−^ mice (Fig. 3b). In WT mice, there were fewer NeuN^+^ cells 15 days post-TBI than at 30 days post-TBI. In contrast, there were more NeuN^+^ cells in CX3CR1^−/−^ mice 15 days post-TBI than at 30 days post-TBI, indicating neuronal cell loss over time. Comparison of genotypes indicated significant differences in numbers of NeuN^+^ cells such that there were more NeuN^+^ cells in CX3CR1^−/−^ mice 15 days post-TBI and more NeuN^+^ cells in WT mice 30 days post-TBI. Collectively, these data demonstrate that CX3CR1 exacerbates cell loss during the acute phase post-TBI (number of neurons is greater in CX3CR1^−/−^ mice 15 days post-TBI than in WT mice) and protects cells during the chronic phase post-TBI (number of neurons is less in CX3CR1^−/−^ mice than WT mice 30 days post-TBI).

### CX3CR1 deficiency correlates with increased microglial proliferation and does not modulate the trafficking of hematogenous myeloid cells to the brain after TBI

**Fig. 4:**
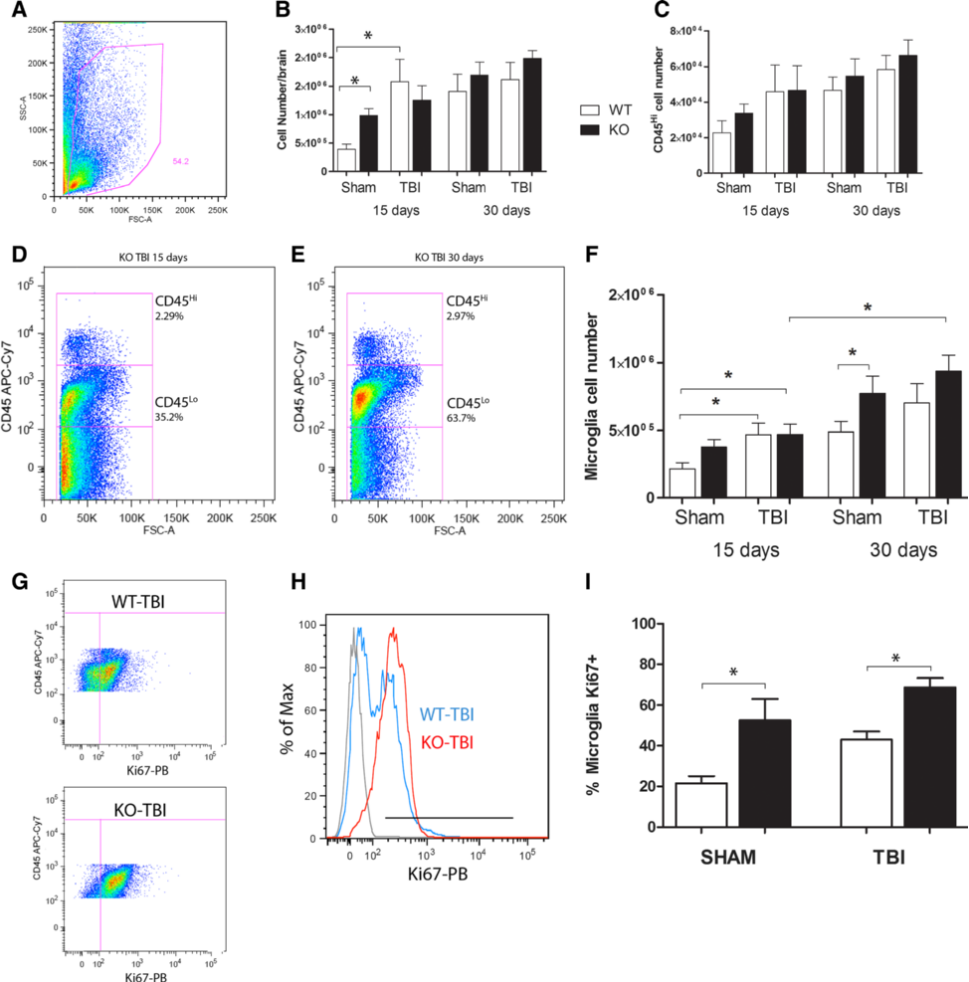
Increased microglial reaction after TBI. CX3CR1^+/+^ (WT; *white bar*) and CX3CR1^−/−^ (KO, *black bar*) brain leukocytes were analyzed by flow cytometry, and total cellularity, numbers of blood derived infiltrating cells, and CD45^Low^ microglial cells were compared. **a–c** After TBI, a significant increase in the overall cellularity was observed when comparing all groups at 15 days (*P < 0.05 comparing sham WT versus WT-TBI 15 days post-TBI), and these changes were sustained 30 days post-TBI. CD45^High^ hematogenous population did not show statistical significance among injured WT and KO mice. **d–f** There was a significant increase in microglial cell number at 15 days post-TBI in WT mice when compared to sham WT group. CX3CR1 KO-TBI group also showed a significant increase in microglia number when comparing 15 and 30 days post-TBI, although effects were also seen in the sham-treated KO group. **g–i** Staining for Ki67 in representative sample of WT-TBI (**g** *upper panel*) and KO-TBI (**g** *lower panel*) groups and also presented in histogram **(h)** with *gray line* showing the isotype controls, WT-TBI Ki67 intensity in *blue line*, and KO-TBI in *red*. Data were quantified **(i)** revealing that in the absence of CX3CR1, microglial cells exhibit a higher proliferative capacity (*P < 0.05)

Because TBI involves infiltration of peripheral blood monocytes into the brain [44], we first isolated mononuclear cells from WT and CX3CR1^−/−^ mice at 15 or 30 days post-TBI and analyzed them with  flow cytometry (Fig. 4a). Total brain mononuclear cells were counted and compared among groups (Fig. 4b; Table 1). After TBI in WT mice, there was an increase in overall cellularity 15 days post-TBI when compared to the sham group (Fig. 4b, *P < 0.05). CX3CR1-KO mice showed a slight increase in overall cellularity at 15 days post-TBI but not statistically significant from the sham group. However, there were no significant differences between injured WT and CX3CR1^−/−^ mice in the CD45^High^ hematogenous population (Fig. 4c), suggesting that overall the contribution of blood-derived cells to the inflammatory process is not altered in CX3CR1^−/−^ mice. Further analysis of the CD45^High^ population was performed using CD11b to identify all myeloid cells, CD11c for dendritic cells (DCs), Ly6G to mark neutrophils, and CD3 to identify T cells. The results did not reveal differences between sham and TBI groups in T cells or infiltrating myeloid cells, including monocytes/macrophages (CD11b^+^CD11c^−^), myeloid derived DCs (CD11b^+^CD11c^+^), conventional DCs (CD11b^−^CD11c^+^), or neutrophils (CD11b^+^Ly6G^+^) (data not shown). Comparison of the CD45^Low^ microglia population between WT (Fig. 4d) and CX3CR1^−/−^ mice (Fig. 4e) reveals a significant increase in both groups when compared to sham controls at 15 and 30 days post-TBI (Fig. 4f). Figure 4d–e shows representative plots, actual numbers are shown on Fig. 4f; (Table 1). There is a significant effect of the sham procedure in the microglia response in CX3CR1-KO mice that is particularly evident at day 30 and significant between the sham WT and sham KO groups. We and others have evidence that the microglia in these mice are more prone to activation in setting of LPS injection, EAE, and MPTP challenge among other models. However, TBI does have a slight effect toward an increased number of microglia at day 30 although not statistically significant. To assess differences in the proliferation of the CNS-resident microglial population, we performed flow cytometry on cells stained with the proliferation marker Ki67 on sham controls and TBI-induced WT mice (Fig. 4g) and CX3CR1^−/−^ mice (Fig. 4h) 30 days after injury (Fig. 4g–i). CX3CR1^−/−^ mice exhibited a higher proportion of Ki67+ microglia (Fig. 4i) irrespective of injury. This result highlights the importance of CX3CR1 in controlling microglial proliferation even under conditions of skull exposure without CCI because microglial proliferation in naïve (unmanipulated) CX3CR1^−/−^ mice is similar to that of WT mice [43]. Collectively, these results suggest that CX3CR1 plays an important role in inhibiting the microglia reaction in this model of TBI.

### Impact of mild TBI on microglia phenotypes

**Fig. 5:**
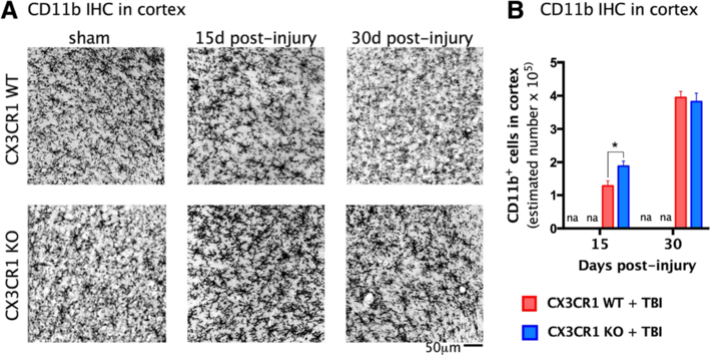
Stereological quantification of CD11B^+^ cells in the cortex at 15 and 30 days post-injury. **a** ×10 magnification micrographs of CD11B staining in the cortex in the vicinity of the lesion. *Upper panel* shows the staining’s intensity in CX3CR1 sham WT and CX3CR1 WT-TBI at 15 and 30 days post-injury. *Lower panels* show the staining’s intensity in CX3CR1 sham KO and CX3CR1 KO-TBI at 15 and 30 days post-injury. **b** The cortical expression of CD11b^+^ cells was significantly increased in CX3CR1 KO-TBI (*n* = 6) mice compared to CX3CR1 WT-TBI mice (*n* = 5) at 15 days post-TBI (*p = 0.05). Two-way ANOVA revealed that the number of CD11b^+^ cells increased in the cortex from day 15–30 post-TBI to the same extent in CX3CR1 WT-TBI and in CX3CR1 KO-TBI mice (^±±^ *p* = 0.001). Staining in sham animals was not determined (nd). CX3CR1 sham WT (*white bar*), CX3CR1 sham KO (*green bar*), CX3CR1 WT-TBI (*red bar*), CX3CR1 KO-TBI (*blue bar*)

To determine whether the protective and neurotoxic effects of CX3CR1 signaling following mild TBI were due to activation of different microglia phenotypes, we quantified microglia immunoreactivity in cortical sections obtained from mice 15 and 30 days post-TBI. We first analyzed CD11b, a marker common to all microglia phenotypes, and found there were more CD11b^+^ cells in CX3CR1^−/−^ mice than in WT mice 15 days post-TBI. By 30 days post-TBI, the number of CD11b^+^ cells increased to the same extent in WT and in CX3CR1^−/−^ mice (Fig. 5 a, b). We performed immunostaining for both sham groups, CX3CR1 KO and WT. CD11b staining intensity showed similar intensity in the cortex of KO and sham WT animals, and it appeared to be lower compared to TBI groups. Therefore, we did not perform stereological analysis for sham animals.

**Fig. 6:**
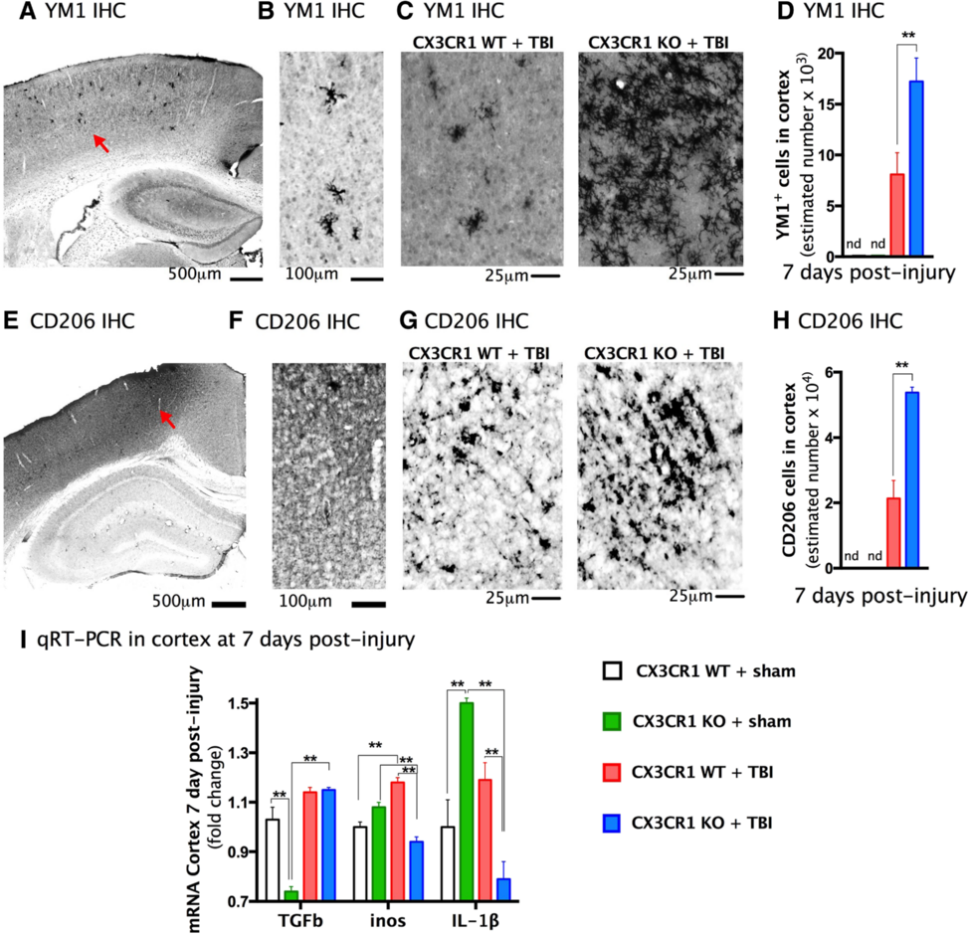
Immunostaining, stereological quantification, and qPCR analysis of M2 and M1 polarization markers in cortex at 7 days post-TBI. **a**–**d** YM1 shows a strong increase in staining in the CX3CR1 KO-TBI compared to CX3CR1 WT-TBI at 7 days post-injury. **a** Micrograph at ×4 magnification showing YM1 staining in the proximity of the lesion area in CX3CR1 KO-TBI mice. **b** Higher magnification (×10) of the area indicated by the *red arrow* in **(a). c** Higher magnification (×20) of the area in the proximity of the lesion of CX3CR1 WT-TBI and CX3CR1 KO-TBI showing in more detail microglial YM1 morphology. **d** Unbiased stereology revealed a significant increase in the number of YM1 and CD2O6^+^ cells (*right panel*) in the cortex of CX3CR1 KO-TBI mice (*n* = 3) compared to CX3CR1 WT-TBI (*n* = 4). **e**–**h** CD206 shows a strong increase in staining in the CX3CR1 KO-TBI compared to CX3CR1 WT-TBI at 7 days post-injury. **e** Micrograph at ×4 magnification showing CD206 staining in the proximity of the lesion area in CX3CR1 KO-TBI mice. **f** Higher magnification (×10) of the area indicated by the *red arrow* in **(a). g** Higher magnification (×20) of the area in the proximity of the lesion of CX3CR1 WT-TBI and CX3CR1 KO-TBI showing in more detail microglial CD206 morphology. **h** Unbiased stereology revealed a significant increase in the number of CD2O6^+^ cells in the cortex of CX3CR1 KO-TBI mice compared to CX3CR1 WT-TBI. YM1 and CD206 were not detected in sham mice (**p < 0.001). **i** Relative mRNA levels of M2 marker TGFβ were significantly lower in sham CX3CR1 sham KO (*n* = 5) compared to CX3CR1 sham WT (*n* = 5). Following TBI, the expression of TGFβ significantly increased in CX3CR1 KO mice compared to sham (*n* = 5; **p < 0.001) but was not different compared to CX3CR1 WT-TBI (*n* = 5). M1 markers iNOS (*middle panel*) and IL-1β (*right panel*) were similar in the cortex of CX3CR1 sham KO (*n* = 5) compared to CX3CR1 sham WT (*n* = 5). Following injury, iNOS and IL-1β mRNA levels in CX3CR1 KO-TBI (*n* = 5) were significantly decreased compared to CX3CR1 WT-TBI (*n* = 5). **p < 0.001). CX3CR1 sham WT (*white bar*), CX3CR1 sham KO (*green bar*), CX3CR1 WT-TBI (*red bar*), and CX3CR1 KO-TBI (*blue bar*)

**Fig. 7:**
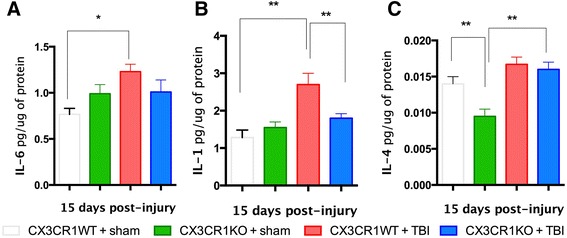
**a**–**c** Fifteen days post-injury, CX3CR1 WT mice show increased protein levels of IL-1 **(a)**, IL-6 **(b)**, and decreased levels of IL-4 **(c)**. CX3CR1 KO-TBI mice show increased IL-4 protein levels. CX3CR1 sham WT (*white bar*), CX3CR1 sham KO (*green bar*), CX3CR1 WT-TBI (*red bar*), and CX3CR1 KO-TBI (*blue bar*) (*P < 0.05)

**Fig. 8:**
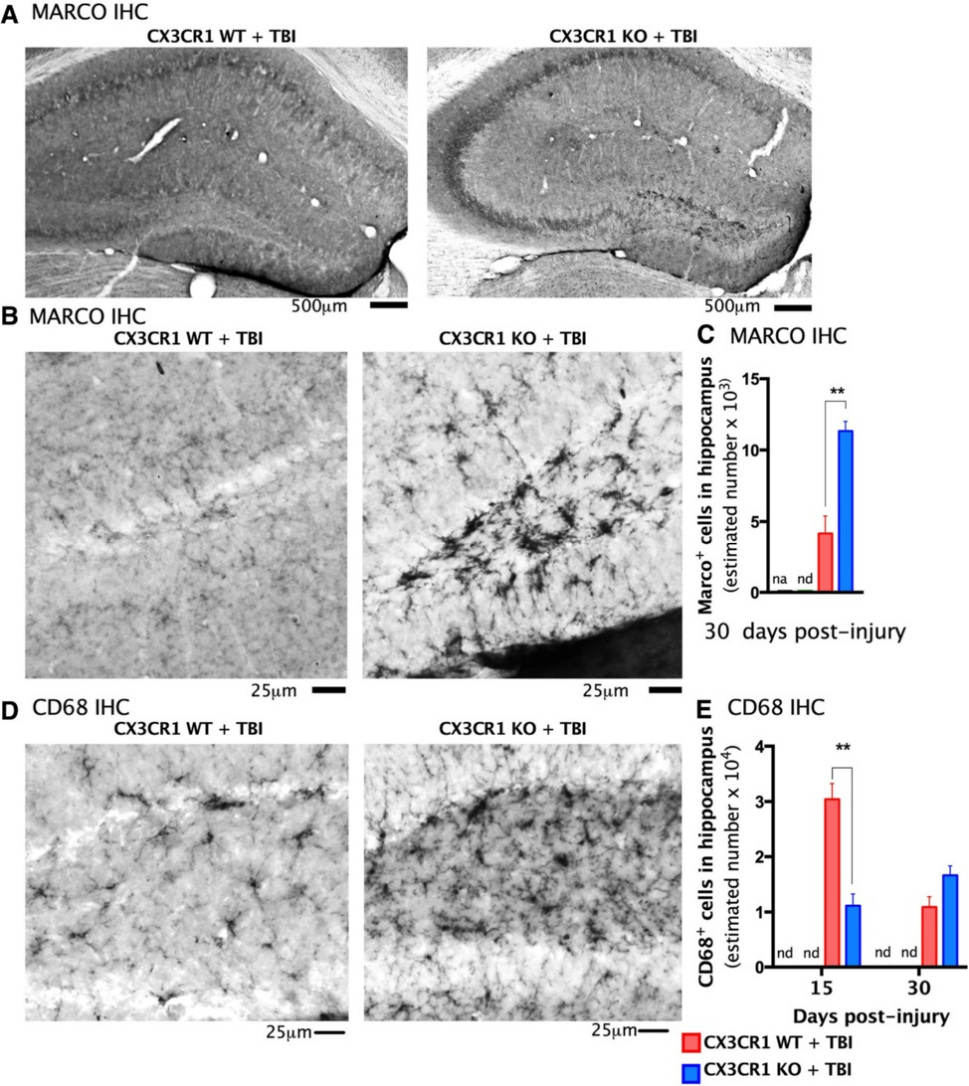
Immunostaining and stereological quantification for M1 polarization markers in hippocampus at 30 days post-TBI. **a** Micrograph at ×4 magnification showing Marco staining at 30 days post-TBI in the dentate gyrus of CX3CR1 WT-TBI and CX3CR1 KO-TBI mice. **b** Higher magnification (×20) of the dentate gyrus of CX3CR1 WT-TBI and CX3CR1 KO-TBI showing in the hippocampus show a strong increase in staining in the CX3CR1 KO-TBI compared to CX3CR1 WT-TBI at 30 days. **c** Unbiased stereology quantification of Marco revealed a significant increase in the number of Marco^+^ cells in the hippocampus of CX3CR1 KO-TBI compared to CX3CR1 WT-TBI (*p* < 0.001). **d** Micrograph at ×20 magnification of the dentate gyrus of CX3CR1 WT-TBI and CX3CR1 KO-TBI showing in the hippocampus show a strong increase in CD68 staining in the CX3CR1 KO-TBI compared to CX3CR1 WT-TBI at 30 days post-TBI. **e** Time course of CD68 expression at 15 and 30 days post-injury. Stereological quantification of CD68^+^ cells revealed a significant decrease in CX3CR1 KO-TBI mice compared to CX3CR1 WT-TBI at 15 days post-TBI. Although the difference did not reach statistical significance, at 30 days post-TBI, expression of CD68 was higher in CX3CR1 KO-TBI mice compared to wild-type (**p < 0.001)

Then, we immunostained cortical tissues for markers associated with M2 microglial and M1 microglial activation phenotype and at 7, 15, and 30 days post-TBI (YM1 chitinase 3-like 3; CD206 mannose receptor; CD68 Marco). At 7 days post-TBI, the expression of the M2a markers YM1 and CD206 increased in the cortex of brain-injured CX3CR1^−/−^ mice, but not brain-injured WT mice (Fig. 6a–h showing M2 expression in the cortex). YM1 and CD206 were not detected in the brains of sham animals of either genotype (Fig. 6d, h). No M2 markers were detected at 15 or 30 days post-TBI. We then used qRT-PCR to determine M2-TGFβ-associated gene expression at 7 days post-TBI. Sham CX3CR1^−/−^ mice had decreased TGFβ mRNA levels (*p* < 0.001) compared to sham WT mice (Fig. 6i). Seven days post-TBI, TGFβ mRNA significantly increased in injured CX3CR1^−/−^ mice as compared to sham CX3CR1^−/−^ (*p < 0.001).* No difference was observed between injured CX3CR1^−/−^ and injured WT mice. We also analyzed gene expression of iNOS and IL-1β at 7 days post-TBI. Levels of mRNA for iNOS and IL-1 were significantly greater in sham CX3CR1^−/−^ mice than sham WT mice (Fig. 6i, *p* < 0.001). In contrast, iNOS and IL-1 significantly decreased in injured CX3CR1^−/−^ mice compared to injured WT mice. Furthermore, iNOS and IL-1 mRNA levels in injured CX3CR1^−/−^ mice were significantly decreased compared to sham CX3CR1^−/−^ mice (see Table 2). Finally, we analyzed M1 and M2-associated protein expression of IL-1β, IL-6, and IL-4, respectively, in CX3CR1^−/−^ and WT mice at 15 days post-TBI. Figure 7 shows that IL-1 and IL-6 increased in WT mice subjected to TBI, whereas IL-4 increased in CX3CR1^−/−^ mice. M1 marker Marco was analyzed at 30 days post-TBI, and M1 marker CD68 was analyzed at 15 and 30 days post-TBI (Fig. 8). The time course and anatomical expression of Marco was different between WT and CX3CR1^−/−^. In WT mice, Marco was expressed in the hippocampus (but not in the cortex) at 7, 15, and 30 days post-TBI, while in CX3CR1^−/−^ mice, Marco expression was only evident at 30 days post-TBI; thus we only quantified Marco at 30 days in hippocampus. Thirty days post-TBI, Marco was highly expressed in injured CX3CR1^−/−^ mice (Fig. 8a–c). CD68, a specific marker of phagocytic microglia, was decreased at 15 days post-TBI in CX3CR1^−/−^ mice relative to WT mice, but by 30 days post-injury, there were no significant differences between genotypes (Fig. 8d, e).

**Table 2:** CT, ΔCT, and fold change. TGFβ, iNOS, and IL-1β raw numbers as plotted in Fig. 6g. Numbers are expressed as CT, ΔCT, and fold change (means and standard deviations) normalized against wild-type sham

Treatment	Genotype	Gene	CT (mean ± SD)	ΔCT (mean ± SD)	Fold change (mean ± SD)
Sham	WT	TGFβ	25.368 ± 0.31	12.429 ± 0.31	1.000 ± 0.20
	KO		25.751 ± 0.23	12.775 ± 0.26	0.782 ± 0.14
TBI	WT		25.268 ± 0.03	12.255 ± 0.11	1.107 ± 0.08
	KO		25.451 ± 0.32	12.451 ± 0.33	1.154 ± 0.06
Sham	WT	iNOS	29.156 ± 0.11	16.558 ± 0.11	1.000 ± 0.07
	KO		29.037 ± 0.12	16.450 ± 0.14	1.080 ± 0.11
TBI	WT		28.802 ± 0.24	16.335 ± 0.29	1.187 ± 0.13
	KO		29.168 ± 0.06	16.654 ± 0.08	0.933 ± 0.09
Sham	WT	IL-1β	28.340 ± 1.01	16.747 ± 099	1.000 ± 0.60
	KO		29.705 ± 1.73	17.109 ± 1.72	1.521 ± 0.17
TBI	WT		28.903 ± 0.93	16.435 ± 0.90	1.191 ± 0.57
	KO		29.259 ± 0.80	16.715 ± 0.80	0.968 ± 0.51

## Discussion

Results of this study demonstrate for the first time that CX3CR1 modulates responses to mild TBI in a time-dependent manner. During the acute post-traumatic period (24 h–15 days), neuronal cell loss is greater in the brain-injured WT than in the brain-injured CX3CR1^−/−^ mice. In contrast, during the chronic phase (30 days post-injury), lack of CX3CR1 exacerbates neuronal damage. The acute post-TBI phase in CX3CR1^−/−^ mice is associated with a protective M2 microglia phenotype as evidenced by upregulation of YM1 and CD206 and increased TGFβ, IL-4, and IL-10. Concurrently with the M2 microglia phenotype during this acute post-traumatic period, responses of CX3CR1^−/−^ mice to TBI include a reduction in M1 microglia phenotype markers, such as iNOS and IL-1β. During the chronic post-injury phase, the brains of injured CX3CR1^−/−^ mice show a profound inflammatory response compared to brains of injured WT mice. This post-traumatic chronic response in mice lacking CX3CR1 is characterized by an inflammatory M1 microglia phenotype, as evidenced by upregulation of Marco, CD68, and the inflammatory cytokines IL-1β and IL-6.

One interpretation for the decrease in TGFβ and IL-4 in the sham KO mice is that lack of CX3CR1 leads to a decrease in the M2-type responses in favor of M1-type responses (increased IL-1β). However, future work will be necessary to test this interpretation and to determine if the polarization of M1-type over M2 type responses is cell autonomous or involves a paracrine signaling mechanism.

The predominant M1 response in injured CX3CR1^−/−^ brain is associated with poorer performance in the Morris water maze. We previously demonstrated that lack of CX3CR1 impairs motor and cognitive function [40]. We now show that lack of CX3CR1 during the chronic phase of mild brain injury exacerbates this already impaired cognitive function. However, it is important to take in consideration that both RT-PCR and ELISA data have been obtained from whole brain tissue, not from extracted microglia; therefore, we cannot determine with absolute certainty the cellular source of these signals. Furthermore, it has to be noted that although the statistical analysis indicated significant difference between CX3CR1 WT-TBI and CX3CR1 KO-TBI in mRNA levels for TGFβ, iNOS, and IL1, the physiological significance of such changes may be questionable and bigger changes may be observed at earlier time points.

Collectively, these observations demonstrate that CX3CR1 is both neuroprotective and neurotoxic in brain injury depending on the time post-injury. Importantly, these findings suggest that antagonizing the CX3CL1/CX3CR1 pathway during a critical period after mild TBI may reprogram the brain inflammatory environment from detrimental to beneficial, favoring endogenous neuroprotective or neurorestorative mechanisms. Furthermore, results from this study provide important new knowledge that is relevant to the differential roles of the CX3CL1/CX3CR1 pathway that have been described in a number of pathologies.

Our findings are in agreement with several reports demonstrating in other models that CX3CR1 drives inflammatory responses during the acute period following CNS injury. For instance, in focal cerebral ischemia, Dénes et al. [24] reported reduced damage to the blood-brain barrier, which correlated with a protected phenotype, reduced ischemic volume, fewer apoptotic cells, and better performance in the behavioral test of adhesive tape removal. Similarly, CX3CR1^−/−^ mice are protected from the middle cerebral artery occlusion, with smaller infarct size at early time points after injury, which is associated with an M2 microglial phenotype [26]. In a model of spinal cord injury, CX3CR1^−/−^ mice had more favorable outcome 5 days post-injury, with neuroprotection and functional recovery, than WT control mice [23]. The improved recovery after traumatic spinal cord injury is associated with reduced recruitment of monocytes/macrophages to the site of injury. Collectively, these studies underscore a role for CX3CR1 signaling in response to CNS injury and suggest that mechanisms by which CX3CR1 promotes neuroprotection during the acute phase of brain injury include a protective microglia phenotype and reduced monocyte/macrophage recruitment.

Our data indicate that CD68, a specific marker of phagocytic microglia, is decreased at 15 days post-trauma in CX3CR1^−/−^ mice and, in this present study, tends to increase by 30 days post-injury. Although microglial phagocytosis of dead or dying neurons can be beneficial by preventing the release of pro-inflammatory molecules, under some conditions, such as inflammation, microglia also phagocytize viable neurons, thus contributing to cell death [45]. Intraventricular infusion of mesenchymal stem cells after TBI induces early and lasting acquisition of a protective M2 microglial phenotype that is associated with reduced CD68 immunoreactivity [46]. Based on these observations, we hypothesize that the reduced phagocytic activity in CX3CR1^−/−^ mice could be beneficial to neurons and perhaps explain neuroprotection at 15 days post-trauma on these animals. On the other hand, increased phagocytosis 30 days post-TBI could be detrimental and increase neuronal death. However, CD68 immunoreactivity may not necessarily correlate with phagocytosis [47, 48]. For example, microglia that are phagocytizing apoptotic cells in physiological conditions in the hippocampus do not express CD68 [47, 48]. Experiments to specifically address this issue remain to be conducted. Nevertheless, our data show that a transient protective M2 microglia phenotype with decreased CD68 phagocytic activity manifests early after mild TBI in CX3CR1^−/−^ mice.

The dramatic shift during the chronic phase of brain injury in CX3CR1^−/−^ mice limits the recruitment of M2 microglia and switches the balance toward the M1 phenotype, which contributes to excessive inflammatory responses and exacerbated neuronal damage. Although a shift in microglia phenotype between the acute and chronic phase of TBI has recently been reported [49], we extend these observations by identifying CX3CR1 as a possible regulator of this microglia/macrophage phenotype switch following mild TBI. It is important to note however, that the transition between M1 and M2 microglia phenotypes is not all or none, and several intermediate microglia phenotypes with overlapping features have been described during the shift between alternatively activated phenotypes. Indeed, recently Morganti et al. [50] using CX3CR1^GFP/+^ CCR2^RFP/+^ reporter mice have demonstrated a broad spectrum of M1-M2 polarization mRNA gene expression following TBI, with changes occurring primarily during the first 48 h. Importantly, they reported that there was not a clear delineation of an exclusive M1, M2a, or M2c phenotype. Several factors could explain differences between our study and that of Morganti et al.; ours is a model of mild TBI model, and our M1 and M2 analysis was based on both gene expression and immunohistochemical assessment, whereas the Morganti study only reported on gene expression.

Although our data do not provide information as to precisely how CX3CR1 dictates the recruitment of M1/M2 microglia during acute or chronic stages of the disease, there likely are multiple mechanisms that regulate the neuroprotective vs. neurotoxic effects of microglia after mild TBI. Relative expression of CX3CR1 defines two phenotypically distinct monocyte subsets. Monocytes of the Ly6C^high^/CX3CR1^low^ subset express CCR2, a chemokine receptor that facilitates recruitment of inflammatory monocytes to the site of injury. Conversely, monocytes of the subset Ly6C^low^/CX3CR1^high^ are CCR2 deficient and are dependent on CX3CR1 for recruitment [51–53]. CX3CR1^+^ monocytes may differentiate from Ly6C^high^ monocytes in tissue and thus may not require CX3CR1 for recruitment [54, 55]. These distinct monocyte populations are associated with tissue pathology and repair. For example, in a model of myocardial infarction, tissue repair requires recruitment of Ly6C^high^ and then Ly6C^low^ monocytes [53]. Ly6C^high^ monocytes are proteolitic, degrade injured tissue, and give rise to M1 macrophages in vivo [35, 56]. Other reports demonstrate that the Ly6C^low^/CX3CR1^high^ monocyte subset is associated with beneficial effects as this population is capable of tissue healing [53].

In experimental models of spinal cord injury and focal cerebral ischemia, tissue damage is attenuated and function recovers earlier following injury in CX3CR1-deficient mice, effects attributed to reduced recruitment and/or activation of microglia/macrophages [23, 24, 57, 58]. Inhibiting CX3CR1 at early time points in a model of ischemic brain injury protects neurological function and reduces neuropathology by decreasing CD11b^+^/Ly6C^low^/iNOS^+^ monocyte recruitment, microglia proliferation, and leukocyte infiltration [58]. Similarly, Donnelly et al. [23] showed that abolishing CX3CR1 reduces accumulation of CD11b^+^/Ly6C^low^/iNOS^+^ monocytes, which confers neuroprotection and promotes recovery of function at early time points after spinal cord injury. These benefits are associated with suppressed inflammatory signaling in microglia and microglia-derived macrophages.

In our model of mild focal TBI, we did not detect differences in leukocyte infiltration from the periphery between wild-type and CX3CR1-deficient mice. Lack of differences between CX3CR1-deficient and wild-type mice could be due to the post-TBI time point analyzed and/or the mild TBI induced in this study. However, based on these aforementioned observations and our data, we hypothesize that after focal TBI, CX3CR1 plays a fundamental role in dictating the timing of recruitment of inflammatory monocyte populations relative to the protective monocyte populations, which results in a detrimental and protective microglia phenotype, respectively. Our results may reconcile data obtained from a number of pathologies with respect to whether CX3CR1 is protective or neurotoxic. Taken together, our data suggest that CX3CR1 may be an ideal target for therapeutic intervention in the acute post-traumatic period to delay secondary brain damage.
